# Rationale for Early Detection of *EWSR1* Translocation-Associated Sarcoma Biomarkers in Liquid Biopsy

**DOI:** 10.3390/cancers13040824

**Published:** 2021-02-16

**Authors:** Felix I. L. Clanchy

**Affiliations:** 1 Kennedy Institute of Rheumatology, Nuffield Department of Orthopaedics, Rheumatology and Musculoskeletal Sciences, University of Oxford, Oxford OX3 7FY, UK; felix.clanchy@kennedy.ox.ac.uk; 2 Botnar Research Centre, Nuffield Department of Orthopaedics, Rheumatology and Musculoskeletal Sciences, University of Oxford, Roosevelt Drive, Oxford OX3 7LD, UK

**Keywords:** sarcoma, liquid biopsy, early detection, biomarkers, *EWSR1*

## Abstract

**Simple Summary:**

Tumour cells often spread from the primary site to new tissues in a process known as metastasis. It has been demonstrated that there is a relationship between the number of circulating tumour cells in the blood and the tumour burden in patients with some forms of sarcoma, which is a cancer of bone and connective tissue. Sarcomas have a particular bias towards metastasis via the blood stream, which is able to be sampled relatively non-invasively, and many forms of sarcoma have well-characterised biomarkers that distinguish them from other blood cells. These characteristics potentiate the use of blood for ‘liquid biopsies’ for patients who are in remission, to detect a relapse as early as possible. This review summarises developments for sarcomas that are associated with the translocation of *EWSR1* and similar genes.

**Abstract:**

Sarcomas are mesenchymal tumours that often arise and develop as a result of chromosomal translocations, and for several forms of sarcoma the *EWSR1* gene is a frequent translocation partner. Sarcomas are a rare form of malignancy, which arguably have a proportionally greater societal burden that their prevalence would suggest, as they are more common in young people, with survivors prone to lifelong disability. For most forms of sarcoma, histological diagnosis is confirmed by molecular techniques such as FISH or RT-PCR. Surveillance after surgical excision, or ablation by radiation or chemotherapy, has remained relatively unchanged for decades, but recent developments in molecular biology have accelerated the progress towards routine analysis of liquid biopsies of peripheral blood. The potential to detect evidence of residual disease or metastasis in the blood has been demonstrated by several groups but remains unrealized as a routine diagnostic for relapse during remission, for disease monitoring during treatment, and for the detection of occult, residual disease at the end of therapy. An update is provided on research relevant to the improvement of the early detection of relapse in sarcomas with *EWSR1*-associated translocations, in the contexts of biology, diagnosis, and liquid biopsy.

## 1. Introduction

Haematological tests have been a commonplace method for diagnosing and monitoring a myriad of conditions for many decades. In recent years, liquid biopsies have been developed to overcome limitations in monitoring disease activity in cancer. While tissue biopsies remain the gold standard for the diagnosis of tumours, liquid biopsy offers the advantages of a non-invasive procedure that can be performed repeatedly during and after the course of treatment [1,2]. The most common source of liquid biopsy is the blood, which need only be collected in small volumes (~10 mL), but the downstream analyses are usually compatible with fluid or cells collected from the more invasive bone marrow aspirates [3], fluid collected from ascites [4] or lymphatic vessels [5], urine [6], lavages [7], or cerebrospinal fluid [8], if necessary.

**Figure 1:**
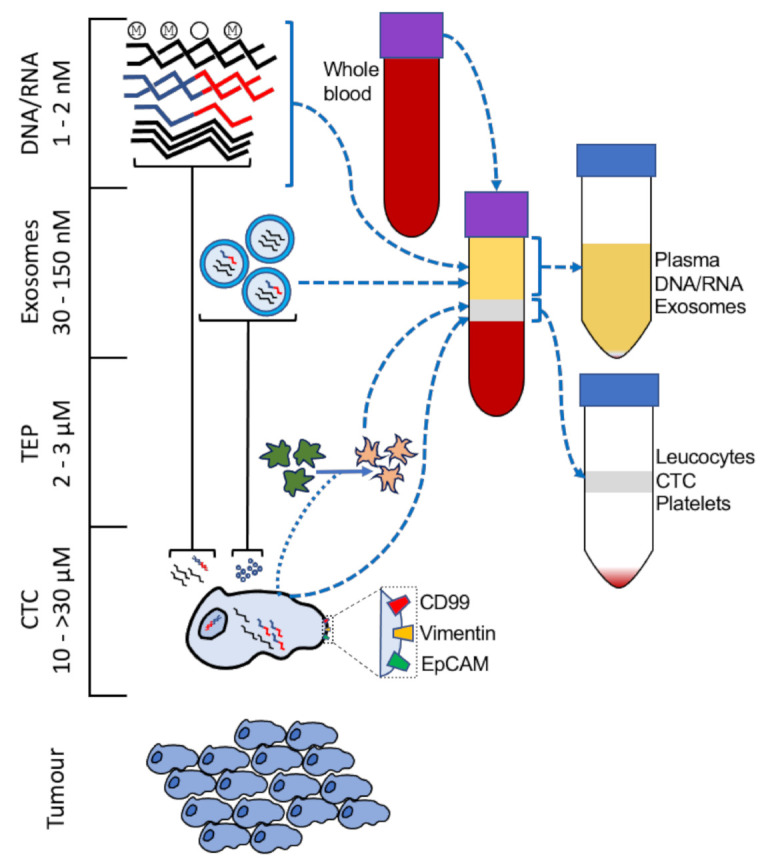
Tumour components present in liquid biopsy. Enrichment of circulating tumour cells (CTC), tumour-educated platelets (TEP), exosomes, and free polynucleotides is achieved by centrifugation and density separation. Whole blood from patients may be centrifuged to separate the plasma layer and the ‘buffy coat’ (leucocytes, platelets, and CTC) from the denser granulocyte/erythrocyte layer. RNA from CTC or cfRNA may be of the fusion gene (e.g., *EWSR1-FLI1* [1]) or, as with TEP [19] and exosomal RNA [20], differentially expressed upon relapse. cfDNA from tumours may be differentially methylated [21] and/or contain chromosomal translocations [22]. Surface markers (e.g., CD99, Vimentin, EpCAM) may be detected by FACS or IHC [23,24,25].

An analysis of the response to treatment by liquid biopsy is complementary to an assessment of primary tumour volume, measured by imaging, during treatment, but the greater value of liquid biopsy is in the period after surgical excision or radiation therapy. As micro-metastases in the tissue fall below the threshold of detection for many imaging techniques, the early detection of relapse offers an opportunity to take ameliorative action in a timely fashion, and the development of assays to monitory disease status by liquid biopsy has been reported for cancers of the breast [9], colon [10], pancreas [11], lung [12], and esophagus [13]. Several forms of liquid biopsy have been developed and are broadly divided into free polynucleotides derived from the tumour (cell-free tumour DNA/RNA, (cfDNA/cfRNA)), anuclear membrane-enclosed vesicles such as tumour cell exosomes or tumour educated platelets, and circulating tumour cells (CTCs) (Figure 1); circulating macrophage-like cells [14], other tumour-associated cells [15], and metabolic pathway enzymes and their metabolites [16,17,18] have also been found to predict prognosis in cancer as well.

cfDNA is an attractive resource for liquid biopsies as, unlike viable cells, samples can be easily frozen for short term preservation after the fractionation of the blood, and well-established protocols exist for the extraction of circulating DNA from plasma. In addition, the analysis of oncogenic mutations measured by PCR, tumour associated DNA methylation [21], and DNA-nucleosome complexes [26] can be detected and quantified. The epigenetic landscape of sarcoma tumours has been explored in several studies [27,28,29,30], and, although a consensus on epigenetic targets, therapies, and biomarkers in sarcoma is yet to emerge, recent perspectives on this area of research have attempted to refine the progress made so far [31,32,33]. In the context of liquid biopsy, several methylation sites have been described as predictive of prognosis for colorectal cancer [34], breast cancer [35], and liver cancer [36]. The methylation status of cfDNA can also be predictive for multiple common cancers, allowing population-wide screening of asymptomatic people at risk [37,38]. As a surrogate marker for the presence of cancer and response to treatment, cfRNA is less well studied, but recent findings have refined the potential use of plasma-derived mRNA [1,39] and miRNA [40]. The relatively greater stability of miRNA offers practical advantages over other forms of RNA, particularly mRNA, due to the effects of temperature and endogenous RNases [40].

Many cell types generate exosomes as part of normal physiological processes to facilitate intercellular communication, and under different states of activation the rate of generation and composition changes [41]. The exosome functions as an extension of the source cell and contain proteins [42] both embedded and within the enclosing lipid bilayer, as well as polynucleotides [43] that modulate the target cell with which it fuses. Research into the function of small extracellular vesicles (30~150nm) in the processes that potentiate local invasion [44], pre-metastatic niche formation [45], and immune system modulation [46] has provided insight into the distinguishing characteristics of tumour-associated exosomes that make them useful for liquid biopsies (reviewed in Zhou, B et al. [20]). Platelets are 20–30 times larger than the largest exosomes and are capable of releasing exosomes of their own, and current research indicates tumours change the platelet RNA profile to a degree distinguishable from normal patients [19]; recent results for proof-of-concept screening in sarcoma patients indicate substantial differences compared to healthy donors and sarcoma patients in long-term remission [47].

CTC are shed from the tumour and are responsible for the development of metastases. Despite the discovery of tumour cells circulating in the blood in the 1860s [48], methods for their isolation and detection have been developed for clinical application only in the last two decades. Estimates for the prevalence of CTC range from 1–10 per mL of blood [49]. For a typical adult this equates to an upper total estimate of 50,000 CTC at any time, suggesting that the odds of any particular CTC becoming a metastasis are very low. The size of tumour cells varies according to the tissue of origin and differentiation state, ranging from pancreatic cancer cells (~12 μm) to non-small cell lung cancer cells (>30 μm) [50,51].

## 2. *EWSR1* in Health and Disease

*EWSR1* is a highly conserved gene encoding a multi-domain protein (EWS) that is expressed in many cell types. While much of the understanding of *EWSR1*/EWS centres around the chromosomal translocation that causes sarcoma, naturally occurring variants and transgenic mice indicate the diverse roles played this molecule (and closely related genes *FUS* and *TAF15*) and highlight challenges for therapeutic targeting in sarcoma. Mutations in *EWSR1*, *FUS,* and *TAF15* have been associated with a minor subset (≥5%) of neuro-degenerative disorders including amyotrophic lateral sclerosis and fronto-temporal dementia [52]. Mice without *EWSR1* are stunted at birth and depending on genetic background are unlikely to survive to maturity [53]. A combination of in vivo and in vitro studies have demonstrated that disruption of *EWSR1* potentiates a reduction in the number and activity of mitochondria [54], dysfunctional gametogenesis, and neuronal atrophy, resulting in motor function deficits [52,55]. Evidence of premature cellular aging in vivo may be contributed to by dysregulated autophagy, which is observed in *EWSR1*^–/–^ cells [56]. 

*EWSR1* has a multifunctional role in transcriptional regulation and RNA splicing and affects multiple cellular pathways. New functions and affected pathways continue to emerge however, the interaction with the basal transcription factors Transcription factor II D (TF_II_D) [57] and CREB-binding protein (CBP) [58], and in turn RNA polymerase, indicate a central role in during transcription; a post-transcriptional splicing role has also been demonstrated for mRNA and miRNA [59].

### 2.1. EWSR1 Translocations Are Common in Sarcoma

**Figure 2:**
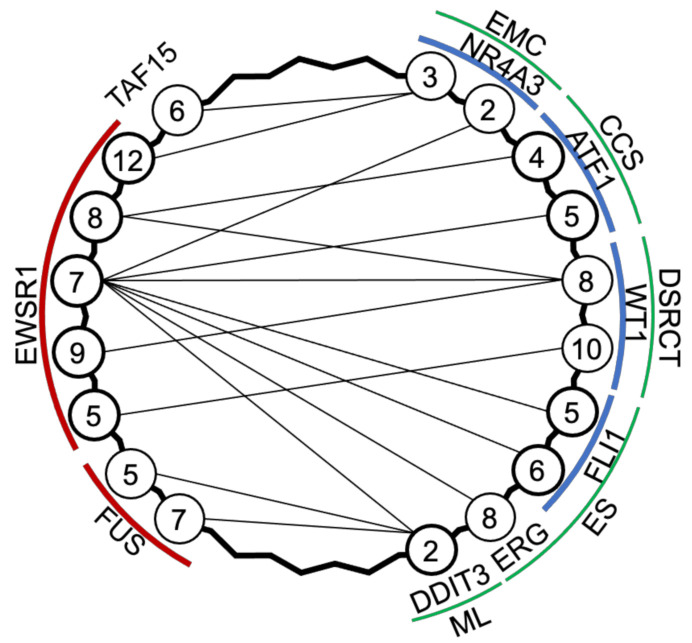
Translocation partners in EWSR1-associated sarcomas. Extraskeletal mxyoid chondrosarcoma (EMC) [64,65,66], clear cell sarcoma (CCS) [67], desmoplastic small round cell tumour (DSRCT) [68], Ewing’s sarcoma (ES) [69], and myxoid liposarcoma (ML) [70,71] have common variants, which are driven by genes *EWSR1*, *FUS*, *TAF15* fused to *NR4A3*, *ATF1*, *WT1*, *FLI1*, *ERG,* or *DDIT3*; numbers refer to exons adjacent to the mRNA splicing.

While the involvement of similar *EWSR1*-associated gene translocations in several forms of sarcoma presents a challenge when distinguishing between subtypes of a particular sarcoma, in the context of the cell of origin, the differing cell phenotypes resulting from each variant permit the determination of the effect of each mutant gene. Angiomatoid fibrous histiocytoma, primary pulmonary myxoid sarcoma, and myoepithelial tumours of soft tissue are exceptionally rare forms of sarcoma that harbour translocations of the *EWSR1* gene (*EWSR1*-*CREB*, *EWSR1*-*ATF1*) or the *FUS* gene (*FUS*-*AFT1*). Despite their rarity, an emerging pattern is of non-aggressive tumours that may recur locally after excision but infrequently metastasize. The greater need for ongoing surveillance for *EWSR1*-associated sarcomas lies with the more aggressive and malignant Ewing’s sarcoma (ES), desmoplastic small round cell tumour (DSRCT), clear cell sarcoma (CCS), myxoid liposarcoma (ML), and extra-skeletal myxoid chondrosarcoma (EMC) (Figure 2). Although these sarcomas have variable cell phenotypes, transcriptomes, prevalence, and prognoses, they are all driven by *FUS*, *EWSR1*, or *TAF15* (sometimes referred to as the ‘FET’ gene family) fused to a transcription factor. As discussed below, although the differing tissues of origin (where known) may present challenges for diagnosing, classifying, and treating these sarcomas, their unifying trait of well-characterised gene fusions presents an opportunity when developing assays for liquid biopsies. 

### 2.2. Ewing’s Sarcoma

Ewing’s Sarcoma (ES) is a highly malignant tumour that occurs in the bone or surrounding tissue, mainly in young people, and while rare is one of the more common forms of sarcoma. The diagnosis of ES is confirmed after a cytogenetic analysis of the chromosomal translocation using FISH break apart probes; the expression of CD99 by IHC can also confirm diagnosis [60], as can next generation sequencing (NGS) where necessary [61]. Metastases usually occur in the lung and are present in ~25% of patients at diagnosis [62].

The treatment options for ES have not improved for several decades and although overall survival rates are as high as 70%, the rate for several patient subgroups (older teens, metastatic at diagnosis, pelvic/rib/spine ES and recurrent ES) is lower; survival rates have remained static for the last three decades [63]. Currently the surveillance options for treated ES patients who are apparently disease free are chest X-ray, blood test for lactate dehydrogenase, and vigilance for bone pain; these methods are neither particularly sensitive nor specific for ES and require improvement. 

Unlike the more common osteosarcoma, ES cells have a genomic translocation giving rise to well characterised mutant fusion genes such as *EWSR1-FLI1* or *EWSR1-ERG*, which primarily drive the disease. While the chromosomal translocation point within the introns of these genes is variable [72], ~85% of ES tumour cells express mRNA transcripts containing the exons 1–7 of *EWSR1* fused to the last four or five exons of *FLI1*; ~10% of cases have the *EWSR1-ERG* fusion of exons 1–7 of *EWSR1* and from the sixth exon onwards of *ERG* [69]. The small number of remaining mutations (<5%) typically comprise fusions of *EWSR1-FLI1* or *EWSR1-ERG* at other exon junctions or *EWSR1-ETV1*, *EWSR1*-*FEV,* and *FUS-ERG* translocations [62]. As more variants that produce ES have been identified and characterised, the overall pattern is of *FUS*, *EWSR1*, or *TAF15* usually fused to an ETS family transcription factor. The phenotypes arising from the more common *EWSR1* translocation partners (*FLI1* and *ERG*) are clinically similar [73], although there may be slightly more overall survival in patients with the *EWSR1*-*FLI1* variant compared to all others [74]. In contrast, the rare Ewing-like sarcoma family of tumours, where a FET gene family member fuses to non-ETS family translocation partners (*NFATC2*, *PATZ*, *VEZF1*), has notable differences in the age of incidence [74].

The aberrant protein is primarily responsible for the pathology, with few other mutations consistently observed in ES tumours. Although involvement of EGFR has been demonstrated, and its effect is evident in differences in prevalence correlated with gene variants associated with ethnicity [75], therapies directed towards EGFR have yielded disappointing results [76]. Similarly, IGFR activity potentiates ES development (due in part to autocrine IGF-1-mediated activity [77]), but treatment targeting ligation of IGF-1 to the receptor or the consequent receptor kinase activity yielded poor results [78]. Currently, standard treatment consists of adjuvant chemotherapy, radiation, and surgical excision as appropriate. 

Despite the derivation from tumours of multiple immortalised ES cell lines for each of the most common translocation variants, many avenues of research have terminated in translational cul-de-sacs. More recently the modification by the mutant protein of the epigenome has demonstrated the complexity and heterogeneity of cellular dysfunction yet to be solved in order to develop better treatments [27]. ES is regarded as an immunologically ‘cold’ tumour and, where present, infiltrating immune cells are associated with prognosis, depending upon leucocyte subset [79,80]. Uncertainty regarding the cell type in which ES develops is an impediment to the development of an animal model that reciprocates the human disease. Many cell lines have been derived from patient tumours, but a consensus for the animal model is yet to emerge, with xenografts in immunocompromised mice being the most common approach [81].

As ES CTC tend to spread via the vasculature, and as ES is relatively more common than other forms of *EWSR1* translocation-associated sarcoma, several studies to monitor disease activity have been undertaken using different forms of liquid biopsy. These studies include flow cytometry to detect cells expressing the high levels of CD99 found on the cell surface of ES cells [24], which was investigated by Dubois SG et al., where different FACS antibody panels were compared to distinguish tumour cells from leucocytes. In this study the level for determining a distinct population of CTC by FACS was between 1 CTC per 1 x 10^5^ and 1x10^6^ leucocytes [24]. In a study by Benini S et al., in addition to evaluating methods of CTC isolation, the threshold of detection by qPCR and dPCR was compared, and by the latter method, detection was robust at 1 CTC per mL; this research followed on from a much earlier proof-of-principle study demonstrating PCR detection of CTC in blood and bone marrow, by West DC et al. [3]. The prognostic application of PCR detection of CTC in blood and bone marrow in ES by Schleiermacher G et al. determined a positive correlation between CTC levels (as determined by PCR) and tumour volumes, as well as the risk of relapse [82]. In a pre-clinical murine model of ES, microvesicles containing fusion gene mRNA were detectable in the blood [83]. The detection of cfRNA *EWSR1-FLI1* fusion transcripts by Allegretti M et al. demonstrated that CTC isolation may not be necessary, although no direct enumeration was made of CTC or cfDNA for comparison [1]. Hayashi et al. developed personalised PCR assays for patients using material from the primary tumour and then measured cfDNA during treatment by droplet dPCR [84]; comparative studies in a murine xenograft model demonstrated a correlation between tumour size and cfDNA [84]. Krumbholz et al. also used personalised assays for the detection of cfDNA in ES patients and found a correlation between cfDNA levels and tumour volume throughout treatment [22]. The approaches used in these studies to detect early relapse and monitor disease activity are generally applicable to most forms of sarcoma featuring chromosomal translocation.

### 2.3. Desmoplastic Small Round Cell Tumour

DSRCT is a rare soft tissue sarcoma that occurs in children and adolescents. DSRCT shares with ES uncertainty regarding the cell of origin and involvement of the *EWSR1* gene as a partner in genomic translocation. In the place of *FLI1*, *ERG,* or another ETS family member, *EWSR1* is partnered with *WT1*, with the most common exon junction between the respective genes being 7/8 (see Figure 2) [68]. Due to the similarity to downstream effects of ES fusion proteins, treatment strategies are similar, although average three year survival rates are lower [85,86] and five year survival is as low as 15% [86].

Unlike ES, DSRCT usually manifests as intra-abdominal tumours, which metastasize via lymphatic or blood vessels to lungs, liver, and bone. Although the future diagnosis and therapeutic targeting of DSRCT is aided by the characterisation of the aberrant transcriptome and surface marker screening, the current diagnosis requires confirmation of histological examination by FISH and RT-PCR [87].

DSRCT cells express surface markers from different cell lineages including epithelial (cytokeratin, epithelial membrane antigen), mesenchymal (punctate desmin expression, vimentin), and neuronal (neuronal enolase, synaptophysin) [88]; CD99 is present in a minor number of cases (~25%) [88]. The T cell-suppressing immune check-point molecule CD276 (B7-H3) is highly expressed and may contribute to the immunoregulatory tumour milieu [89].

The protein translated from *EWSR1-WT1* lacks the repressor region normally found in WT1 and leads to upregulated expression of receptors with mitogenic potential (IGF1R and EGFR) and transcriptional regulators (N-MYC, c-MYC, and PAX2-2) [90,91]. The expression of the growth factors VEGFA (and its receptor VEGFR-2) and PDGFα have been shown to be important in a xenograft explant model and in vitro, respectively. The study of DSRCT has been hampered by the lack of tumour-derived cell lines with only one established so far (JN-DSRCT-1), which harbours one of the less common translocation variants; nevertheless, this cell line displays exosomal similarities to DSCRT explants [89].

The potential use of liquid biopsies for disease monitoring was recently demonstrated by Shukla et al., where a personalised assay was developed after NGS genomic sequencing of tumour to determine the translocation breakpoint; cfDNA was subsequently isolated for droplet dPCR or targeted NGS [92]. Prior to this, Colletti M et al. analysed exosomal miRNA from the plasma of DSCRT patients and identified a panel of miRNA sequences not expressed in healthy controls [93].

### 2.4. Clear Cell Sarcoma of Soft Tissue

In contrast to the poorly differentiated phenotype of ES and DSRCT, CCS has distinct features of melanocytic differentiation. As with other sarcomas, each case of CCS is primarily driven by a single mutation particular to each tumour and for CCS genomic translocation results in the fusions of *EWSR1* with *ATF1* or, rarely, with *CREB1* (Figure 2) [67]. The related CCS-like tumour of the gastrointestinal tract tends to express *EWSR1*–*CREB1* with a minor subset of patient tumours expressing *EWSR1*–*ATF1* [94].

The tumour often presents as a slow-growing mass in the deep tissue of the lower extremities. The *EWSR1*–*ATF1* protein can bind to and activate the melanocyte-specific, microphthalmia-associated transcription factor (MITF) via another transcription factor (SOX10) to induce a melanocyte-like phenotype [95]. Currently treatment is by surgical excision, with chemotherapy or radiation used when clean surgical margins are not achieved or not possible due to the proximity of the tumour to inoperable sites. Radiation and chemotherapy have poor response rates for both the primary tumour and metastases, but cleanly excised tumours and a lack of spread indicates a good prognosis.

In most cases, CCS cells have a strong immuno-histological staining of HMB-45, melan-A, and S100 [96]. The detection of translocation has been confirmed by PCR and sequencing in several studies [97,98,99,100]; however, no major study of monitoring disease activity via liquid biopsy has been reported. As the survival rates for CCS are ~40% at 10 years compared to ~60% at five years [101], long-term liquid biopsy surveillance may improve patient outcomes for this slow-growing tumour.

### 2.5. Myxoid Liposarcoma

As with CCS, liposarcomas appear to approximate a differentiated cell type, in this case the adipocyte phenotype, and myxoid liposarcoma (ML) is the second most common amongst several lipoma subtypes, accounting for up to 20% [102]. The cell morphology of ML tumours further stratifies patients when graded according to the degree of ‘round cell’ liposarcoma cells within the tumour, with greater proportions associated with poorer prognosis. As with CCS, non-metastatic spread and clean surgical excision offer a high chance of five year survival, and for ML this is true even for large (>5cm) tumours [103]. As ML tumours are radiation sensitive, local recurrence can be controlled with treatment after therapy and a reduction in tumour volume prior to surgery may prevent adverse outcomes associated with surgery and potentially tumour removal without limb amputation.

ML is caused in many cases by *FUS*-*DDIT3* and less commonly *EWSR1*-*DDIT3* gene fusion [70,71,104], which is hypothesized to lead to a perturbation of PPARγ activity [105,106]; other forms of liposarcoma (pleomorphic sarcoma and the de-differentiated/well differentiated liposarcomas) have variable gene fusions that rarely involve *EWSR1*/*FUS*/*TAF15* [107]. Although PPARγ signalling has been demonstrated to have an anti-tumor activity by supporting differentiation and suppressing proliferation, ML patients with relatively higher expression of PPARγ had reduced recurrence- or metastasis-free survival [108]. For inoperable, metastatic, and recurrent ML tumours that do not respond to conventional treatments, PPARγ agonists may offer long term suppression of disease activity [105]. Monitoring disease activity for patients in remission or during treatment has been performed using circulating cfDNA [109]. In this study by Braig et al., patient’s tumour DNA was sequenced to determine the chromosomal breakpoint, and for each patient a specific qPCR assay was designed for liquid biopsy.

### 2.6. Extra-Skeletal Myxoid Chondrosarcoma

Amongst the less aggressive forms of sarcoma, extra-skeletal myxoid chondrosarcoma (EMC) has an initial tendency towards indolent growth, with metastases present in a minority (~1/3rd) at first presentation or subsequently; the age of diagnosis is usually in the fourth and fifth decades of life [110]. The rarity of EMC has prevented a strong conclusion to be drawn regarding the responses to chemotherapy, other than most forms produce a range of responses [111]; the best treatment remains excision with wide margins. Expression of vimentin, S100, class III β-tubulin, and micro-tubule-associated protein-2 have been noted, which are suggestive of a mesenchymal cell lineage with neuro-endocrine differentiation [112]. The transformation of the cell is driven by the translocation of the *TAF15*-*NR4A3* or *EWSR1*-*NR4A3* fusion proteins [64,65,66]; however, these findings are yet to be translated to a liquid biopsy. Survival at five years was estimated at >80%, but by 15 years had decreased to ~60%, indicating that long term surveillance, especially after five years post treatment, is necessary [110]. 

## 3. Liquid Biopsy for Sarcoma Surveillance

Metastasis may occur despite complete resection; therefore, the possibility of relapse is cause for continued surveillance using liquid biopsy regardless of complete resection. Furthermore, where resection status is unclear, liquid biopsy may be complementary to further treatment or revisional surgery [113]. Although all methods for liquid biopsy have advantages and disadvantages, for sarcoma the detection of CTC has advantages over other forms of liquid biopsy. While isolating and enriching CTC may present difficulties, due to their rarity, the current inability to easily isolate or substantially enrich cfDNA/RNA that is specifically derived from the tumour means that the extra processing required to debulk CTC of unwanted leucocytes is justified by the enhanced signal to noise ratio when detecting fusion genes by PCR. Exosomes and platelets that are derived from or educated by the tumour, respectively, also lack specific markers that would permit routine enrichment. 

For most forms of cancer, the genomic mutations are not specific and must be determined by DNA sequencing for each individual patient. Genomic heterogeneity within the tumour (and between the primary tumour and CTC) may complicate this process. This issue is less complicated in many forms of sarcoma, as a single chromosomal translocation takes place within defined regions of predictable translocation partners. Despite the narrow range of translocation partners, the chromosomal fusion-point varies between patients by many kilobases and would require individual genomic sequencing and assay design to detect cfDNA (or DNA from CTC).

While a single oncogenic fusion gene commonly found in a sarcoma is the primary reason for the development of a transformed cell and eventual tumour, unlike other forms of cancer, there are usually few other genomic aberrations found consistently across patient cohorts. This apparent lack of heterogeneity at the genomic level is yet to be translated into treatments targeted to each mutation, and the more recent observation of intra-tumoural epigenetic heterogeneity may account for differences in disease progression and the response to therapy [27]. 

Despite a lack of success in the decades-long effort to therapeutically target a relatively small number of a sarcoma-specific gene fusion proteins, the consistency of the mutant mRNA exon boundaries is an opportunity in terms of diagnosis and detection of residual disease that is now being fulfilled.

### 3.1. Polynucleotide Liquid Biopsy for Sarcoma Monitoring in Practice

The detection of fusion genes in extracted DNA and RNA can be performed by traditional RT-qPCR, the increasingly more common digital PCR (dPCR), or by utilizing NGS. While both forms of PCR require prior knowledge of a patient’s particular gene fusion, dPCR partitions the reactions into many individual reactions, which are run in parallel, whereas a traditional RT-qPCR is just one reaction per replicate. Within the RT-qPCR reaction, fluorescence at each cycle is measured, and a threshold cycle (C_T_ or C_q_) is calculated at the point where the reaction is in the exponential phase. In contrast, dPCR determines which partitions have or have not amplified a product and, using a Poisson model, the number of starting molecules can be calculated [114]. The advantage of dPCR is greater sensitivity and reduced need for standard curve for quantification; however, RT-qPCR is currently more economical and more amenable to higher throughput [115]. In comparison to PCR, NGS requires no specific assay to determine if sarcoma-associated genes are present in clinical material. Analyses using NGS may be unbiased and genome-wide or instead focussed on panels of known oncogenes either as an oncogene-focussed adaptation of standard NGS (FusionPlex (Archer) [116] or AmpliSeq Focus panel (Illumina) [117]) or variations based on nuclease protection chemistry (Edgeseq (HTG) [118]).

While PCR-based techniques require stringent handling during processing and nucleic acid extraction to prevent contamination, they are more sensitive compared to techniques such as flow cytometry. To detect cfDNA requires DNA sequencing of each patient’s tumour and a personalised PCR assay designed and produced for each sarcoma patient. While this is technically possible, the current cost and expertise required make it impractical [84]. In contrast, surveillance using only three PCR assays could cover >90% of ES patients when measuring the mRNA transcripts of fusion genes by PCR. Therefore, using PCR, the detection of ES cells at very low prevalence (e.g., 1/1–2 million) is feasible due to the mRNA transcripts being present only in the tumour cells. The detection of free polynucleotides was explored in Allegretti M et al., where cfRNA was isolated from the plasma of ES patients and the presence of ES-associated fusion genes detected by dPCR [1]. In this proof-of-principle study, the sole surviving patient (of six) had no detectable transcripts in the final liquid biopsy, and the non-surviving patients had a distinct but minor population of positive events, as measured by dPCR, at different stages of disease, but unlike Krumbholz et al. [22] there did not appear to be detection of circulating tumour-derived polynucleotides that were detectable prior to conventional imaging techniques.

### 3.2. CTC Liquid Biopsy for Sarcoma Monitoring in Practice

The detection of a rare cell requires an extended series of sampling; in practical terms, this means processing millions of cells to distinguish a real population of CTC. This process is greatly enhanced by techniques such as flow cytometry, wherein cells can be analysed at the rate of thousands per second, or automated image analysis, but is still hampered by the rarity of events; one commercially available system ‘Veridex’ (CellSearch®) has been approved by the U.S. Food and Drug Administration [119]. The technical hurdle of extended measurement can be overcome by enriching the target population within the sample, by amplifying the signal or a combination of both. 

By reducing the number of non-CTC within the sample, the CTC population can be identified with greater confidence. The complete elimination of non-CTC is usually not necessary and would be unhelpful for routine laboratory-based screening, as a small amount of cells requires attentive handling. The means to reduce non-CTC are varied and include the relatively simple lysis of erythrocytes, the removal of granulocytes and erythrocytes by density separation centrifugation, or cross-linking non-CTC for elimination with erythrocytes during centrifugation (e.g., RosetteSep^TM^ depletion, STEMCELL Technologies^TM^). These approaches are relatively routine and can be incorporated into a diagnostic workflow to debulk the liquid biopsy prior to detection of CTC. 

Further enrichment can be obtained by positive selection versus antigens known to be highly expressed on CTC (such as vimentin [120], CD99 [2] or EpCAM [23]), by discontinuous density gradients, or by devices specifically designed for this purpose, although for surveillance purposes this is unnecessary. The cell separation solutions recently described include separation devices based upon different cell size (Parsortix (ANGLE) [121]), filtration systems (CellSeive (Creatv) [122]), or microfluidic sorters (On-Chip Sort (On-Chip Biotechnologies) [123]), which are marketed towards research rather than diagnostic testing. As they have the potential to rapidly enrich CTC, cell separation techniques that separate cells based on cell size, and the devices (filters, strainers, and microfluidic chambers) may be more useful as part of a workflow to isolate relatively untouched CTC for analysis where therapies are not effective, and a better understanding of the tumour phenotype is required for better targeted treatment [124].

Sarcoma cells with a specific trait that distinguishes them from other circulating cells will permit routine screening for the presence of a tumour using the selective amplification of a CTC-specific signal. The most practical method incorporating amplification is RT-PCR, which can be used to detect CTC-specific mRNA transcripts. Although single cell methods of mRNA transcript expression detection exist, such as single cell RNA sequencing or FACS-based RNAscope [125], they are uneconomical or require onerous processing for analysis in comparison to bulk lysis of circulating cells, which have been depleted of non-CTC, for mRNA extraction. Detection of CTC by surface marker expression by flow cytometry is labour-intensive and may not give conclusive results where tumour cells circulate at particularly low levels. 

Currently the prohibitive cost and testing capacity does not allow for whole transcriptome or genome sequencing of the biopsy from each suspicious growth. The decreasing cost of sequencing, combined with diagnostic laboratory automation and integrated bioinformatic analysis, may hasten developments further when patients stratified to treatments by this method have a high chance of a good prognosis. Consequently, a customised liquid biopsy for each patient for post-operative surveillance for many solid tumours remains an aspiration. For sarcoma patients, however, the relatively small number of well-characterized fusion proteins encoded by mRNA with specific mutant exon junctions provides an opportunity to establish protocols and infrastructure for future diagnostic platforms. Furthermore, the tendency for sarcoma cells to circulate in the blood provides a rationale for liquid biopsy, particularly as it relates to the detection of CTC, as opposed to exomes and circulating polynucleotides, as there is currently no method to selectively enrich exomes or DNA/RNA that are derived from tumour cells alone. While CTC circulate at a low frequency, RT-PCR can sensitively and selectively amplify this signal and routine methods exist to boost the signal-to-noise ratio by depleting non-CTC. 

## 4. Conclusions

When considering the most practical options for the development of a routine liquid biopsy for the early detection of relapse in sarcoma, several considerations are necessary (Table 1). In order to detect relapse at the earliest stage, the assay must be sensitive and, in this regard, the ability to enrich CTC has an advantage over polynucleotides, exosomes and TEP. Conversely, the relative ease of isolating cfDNA/cfRNA (and to a lesser extent exosomes) from plasma may result in greater amounts of overall polynucleotides collected notwithstanding dilution by non-specific RNA/DNA, and more rapid and simplified sample processing compared to the isolation of CTC. Methods that require detection of a change in expression of biomarkers (e.g., the changing transcriptome of exosomes or TEP) are more resource-intensive compared to sarcoma-specific assays such as RT-qPCR/dPCR assays for gene fusions; additionally, biomarkers that are present in healthy cells may give false positives (e.g., CD99 surface expression). Currently, the cost and complexity of the development of a PCR assay for each patient is a barrier to the use of DNA-based biomarkers. For liquid biopsy assays that incorporate NGS, the analysis of sequencing data is a non-trivial task compared to PCR or flow cytometry. Overall, for *EWSR1*-associated sarcomas in particular, the sensitivity of PCR in combination with the ability to enrich CTC indicates that liquid biopsies that incorporated these elements have an advantage over other methods.

In the 150 years since the discovery of tumour cells circulating in the blood, the specificity and sensitivity of the assays to detect CTC have improved immeasurably. Regrettably, the options for the treatment for many forms of sarcoma have not improved for several decades. The low incidence of sarcomas is a hindrance to the development and testing of new treatments, which is also frustrated by the difficulty of therapeutically targeting a well-characterized set of fusion proteins unique to each subset of patients. While new treatments, alone or in combination, will take time to demonstrate efficacy, the relatively simpler task of improving the detection of relapse at the earliest possible stage will be accomplished by the use of liquid biopsies. For the more common forms of sarcoma such as ES [22] and alveolar rhabdomyosarcoma [126], recent studies have demonstrated that real-time disease surveillance is possible. The studies discussed were undertaken for many reasons such as disease monitoring, classification of patient groups, and demonstration of proof-of-principle, but all provide information and have broad applicability to the goal of early detection of sarcoma relapse. The development of integrated PCR-based diagnostics for the early detection of relapse of non-sarcoma cancers is achievable dependent upon low-cost sequencing of the tumour genome/transcriptome and bespoke PCR assays for each patient’s liquid biopsy, which is preferably undertaken by automated sample processing. Progress towards establishing the infrastructure for a high throughput diagnostic service may be possible with liquid biopsies from sarcoma patients.

## Figures and Tables

**Table 1:** Practical considerations for the early detection of relapse of sarcoma by liquid biopsy.

	cfDNA —PCR	cfRNA —PCR	cfRNA —NGS	Exosomes —PCR	Exosomes —NGS	TEP —NGS	CTC —FACS	CTC — DNA PCR	CTC — RNA PCR
Enrichment of tumour-derived biomarker practical?	N	N	N	N	N	N	Y	Y	Y
Routine sample processing?	Y	Y	N	N	N	N	N	N	N
Sarcoma specific assay?	Y	Y	N^1^	Y	N^1^	N	Y	Y	Y
Biomarker absent in healthy patients?	Y	Y	N	Y	N	N	N	Y	Y
Individually personalised assays required?	Y	N	N	N	N	N	N	Y	N
Simple sample analysis?	Y	Y	N	Y	N	N	Y	Y	Y
Y/N, yes/no									

^1^ NGS focused panels are not specific to one form of sarcoma but more specific than transcriptome-wide sequencing.
